# NMR Studies of Hetero-Association of Caffeine with di-O-Caffeoylquinic Acid Isomers in Aqueous Solution

**DOI:** 10.1007/s11483-014-9368-x

**Published:** 2014-10-03

**Authors:** Nicola D’Amelio, George Papamokos, Jens Dreyer, Paolo Carloni, Luciano Navarini

**Affiliations:** 1 Department of Structural and Molecular Biology, University College London, Gower Place, WC1E 6BT London, UK; 2 German Research School for Simulation Sciences, GmbH 52425, Jülich, Germany; 3 International School for Advanced Studies, SISSA via Bonomea, 265, 34136 Trieste, Italy; 4 illycaffè S.p.A, via Flavia, 110, I-34147 Trieste, Italy

**Keywords:** Caffeine, Di-O-caffeoylquinic acid isomers, NMR, Caffeine heteroassociation, Density functional theory

## Abstract

Caffeine hetero-association with 3,5-di-O-caffeoylquinic acid, 3,4-di-O-caffeoylquinic acid and 4,5-di-O-caffeoylquinic acid in aqueous solution has been investigated by one-dimensional (1D) and two-dimensional (2D) high resolution ^1^H and ^13^C NMR spectroscopy. Self-association of the di-O-caffeoylquinic acid isomers has been studied as well. Caffeine-di-O-caffeoylquinic acid isomers association constants were measured. The value of the association constant of the caffeine-di-O-caffeoylquinic acid complexes is compatible with previous studies and within the typical range of reported association constants for other caffeine-polyphenols complexes. Structural features of the three different complexes have also been investigated by NMR spectroscopy combined with quantum chemical calculations, and the complex conformation is discussed. Our results show that stacking interactions drive the formation of the complexes and that multiple equilibria are present in the interaction of caffeine with 3,4-di-O-caffeoylquinic acid and 4,5-di-O-caffeoylquinic acid while the complex with 3,5-di-O-caffeoylquinic acid seems to be better defined.

**Electronic supplementary material:**

The online version of this article (doi:10.1007/s11483-014-9368-x) contains supplementary material, which is available to authorized users.

## Introduction

The complex formed between caffeine and chlorogenic acid (5-O-caffeoylquinic acid according to IUPAC nomenclature, [1]), the well known major polyphenolic constituent of green coffee bean, was isolated one century ago by Gorter [2]. Sondheimer et al. [3] determined the equilibrium constant of the complex formation reaction in water by spectrophotometry. The same method has been used by Kappeler et al. [4] to study caffeine as well as other purine alkaloids complexation with chlorogenate. In the 70’s, Horman and Viani [5, 6], on the basis of NMR chemical shift data, proposed that the caffeine-chlorogenate complex might be described as a 1:1 type π-molecular complex stabilized by hydrophobic interations. The caffeine-chlorogenate complexation has been indicated as crucial mechanism to explain the compartmentation of caffeine in *Coffea* plants and the qualitative relationship between caffeine and chlorogenic acid content distribution among wild *Coffea* species [7–9]. Recently, it has been proposed as a possible strategy to develop an analytical method to determine caffeine by means of capillary electrophoresis [10].

**Scheme 1:**
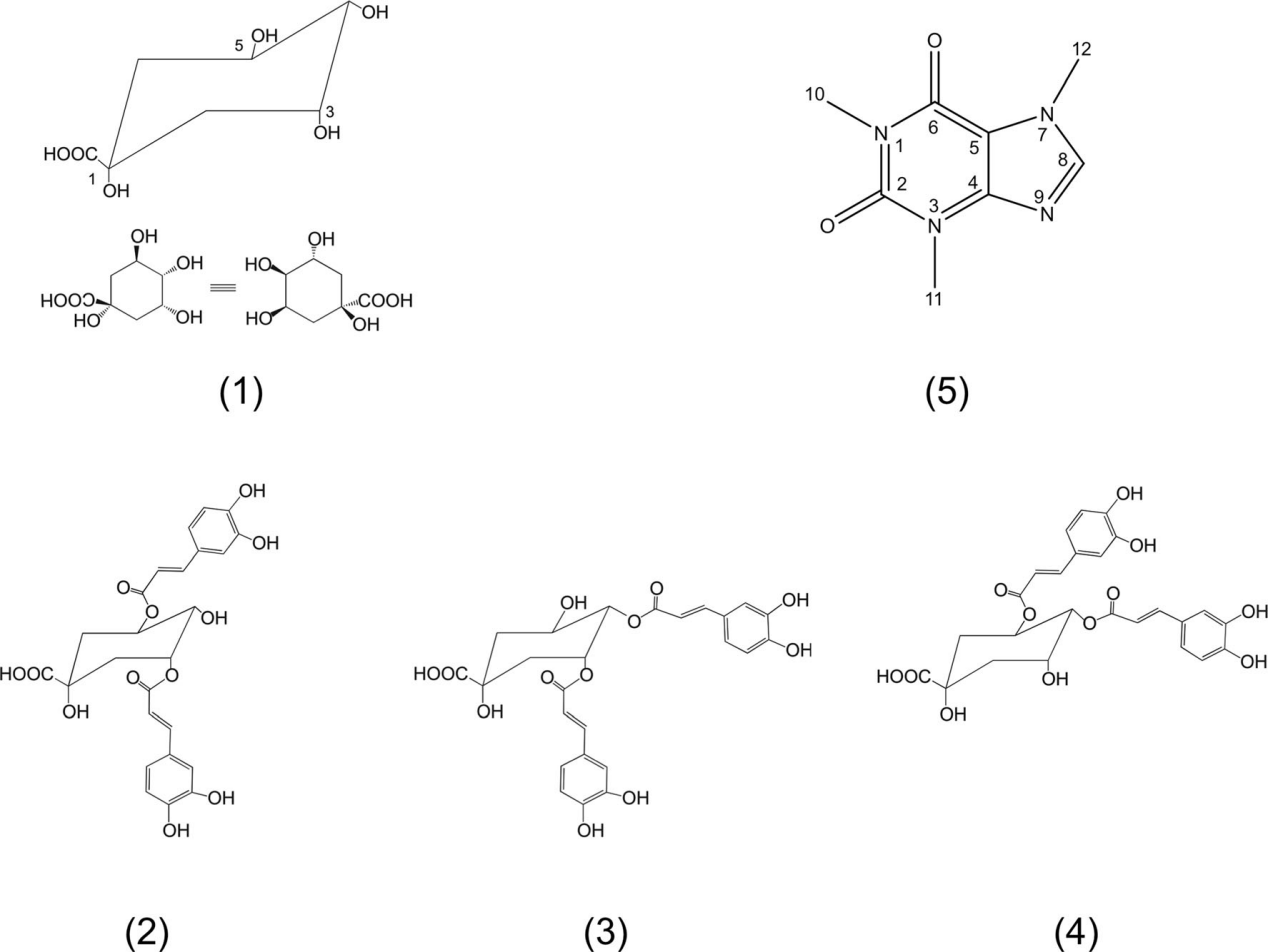
Molecular structures

The caffeine-chlorogenate complex has been re-investigated by high resolution ^1^H-NMR spectroscopy and its structure has been revised [11]. The complex has also been studied in freshly prepared *espresso* coffee brews. Based on the equilibrium constant and the concentration of both complex constituents in water solution, the theoretical molar fraction of bound caffeine can be estimated. In *espresso* coffee, this molar fraction appears lower than expected, suggesting that other species may be involved in the binding of caffeine [11]. The present investigation has been largely stimulated by the hypothesis that other coffee polyphenols could be involved in caffeine binding. In particular, in addition to 5-O-caffeoylquinic acid, many other *trans*-cinnamic (hydroxycinnamic) acids esters with (-)-quinic acid (Scheme 1, according to IUPAC: 1 L-1 (OH),3,4/5-tetrahydroxycyclohexanecarboxylic acid (**1**); name revised in 1997 as 1s_n_,3R,4s_n_,5R-tetrahydroxycyclohexanecarboxylic acid [12]), are present in coffee brews [11].

Di-O-caffeoylquinic acid isomers (3,5-di-O-caffeoylquinic acid (**2**), 3,4-di-O-caffeoylquinic acid (**3**) and 4,5-di-O-caffeoylquinic acid (**4**) in Scheme 1) represent good candidates for possible hetero-association with caffeine, in view of their particular molecular architecture and thanks to their recent availability as commercially available standards. Moreover, these coffee compounds have been already characterized by NMR, albeit not extensively [13–15].

As far as we know it is the first time that self-association and hetero-association with caffeine in aqueous solution of di-O-caffeoylquinic acid isomers have been investigated by NMR spectroscopy. NMR data related to hetero-association with caffeine have been discussed in view of quantum chemistry calculations in order to reveal possible complex conformations.

## Materials and Methods

3,5-di-O-caffeoylquinic, 3,4-di-O-caffeoylquinic and 4,5-di-O-caffeoylquinic acids were purchased from Phytolab GmbH (Germany). Caffeine and all other chemicals were purchased from Sigma Aldrich (Sigma Chemical Corp., St. Louis, MO) and used without further purification. Solutions containing polyphenols were prepared in 80 mM phosphate buffer at pH 7 in order to prevent pH effect on chemical shift at different concentrations. Caffeine and polyphenols were dissolved in H_2_O (10 % D_2_O) containing TSP (sodium 3-(trimethyl-silyl) propionate-2,2,3,3, d4) as internal standard for chemical shift referencing. Samples were at concentration ranging from 1 μM to 20 mM. ^13^C and ^1^H chemical shift assignment reported in Supplementary tables S1-S3 were determined on 20 mM samples.

### NMR spectroscopy

All NMR measurements were performed at 293 K on a Bruker Avance 700 MHz spectrometer. Assignment of di-O-caffeoylquinic acid isomers were accomplished by a series of 2D spectra, namely: 2D-NOESY, 2D-ROESY and ^1^H,^13^C-HSQC.

TOCSY spectra were recorded with a total spin-locking time of 60 ms using a MLEV-17 mixing sequence; ROESY spectra were performed with a standard pulse sequence using a ROESY spin-lock of either 300 or 500 ms. During all 2D experiments, water suppression was achieved by presaturation. NOESY spectra were recorded with mixing times of either 300 or 500 ms.

### Quantum Chemistry

Density Functional Theory (DFT) using the ωB97X-D [16, 17] functional and the cc-pVDZ [18] basis set was used to build up the 3,5-di-O-caffeoylquinic acid (**2**), 3,4-di-O-caffeoylquinic acid (**3**) and 4,5-di-O-caffeoylquinic acid (**4**) complexes with caffeine. The ωB97X-D functional shows good performance among DFT techniques describing the dispersion interactions and therefore the distance dependence of interaction energies in bimolecular complexes [19]. The polarizable continuum model (PCM) with the default integral equation formalism [20] was used to take aqueous solvation of the complex into account. All monomers were first geometry optimized. Calculated ^1^H-NMR and ^13^C-NMR spectra were in good accord with experiment (See SI). Then, the optimized structures were used to construct the initial structures of the complexes. Following the discussion on NMR data for the 3,5-di-O-caffeoylquinic acid, 3,4-di-O-caffeoylquinic acid and 4,5-di-O-caffeoylquinic acid in complex with caffeine, we built the initial structure of each complex by placing caffeine in a stacked position above and below the aromatic rings of each caffeoyl arm.

## Results and Discussion

**Fig. 1:**
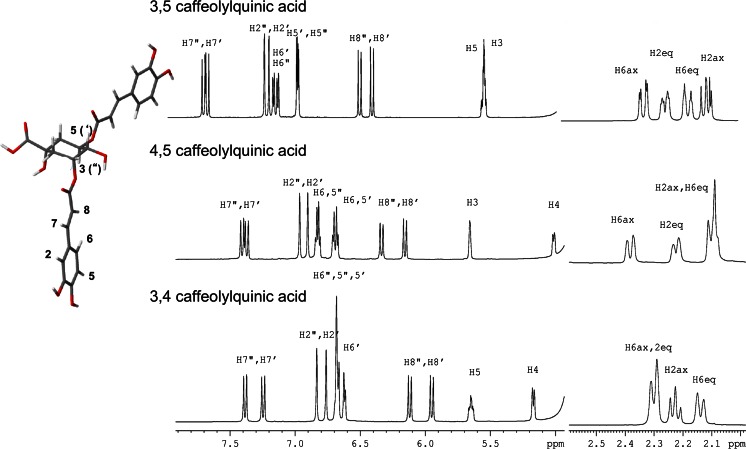
^1^H NMR spectra of 3,5– 4,5- and 3,4-di-O-caffeoylquinic acids. 3,5-di-O-caffeoyl acid is shown in the insert with atom numbering

The NMR spectra of 3,5- 4,5- and 3,4-di-O-caffeolylquinic acid isomers in water solution at pH 7 are reported in Fig. 1. It can be observed that both caffeoyl arms are distinguishable in all three compounds and they were labelled with (‘) and (“) symbols. Although the assignment of each caffeoyl arm to a specific linkage position on the quinic acid moiety is only temptative (based on ROE and NOE cross peaks), substituents linked in position 5 are primed, (‘), while the ones in position 3 are doubly primed, (“) (Fig. 1). In the isomers where these positions are not substituted, the arm can be labeled for comparison (e.g. position 5 in the 3,4-di-O-caffeoylquinic acid is not substituted but since the double prime symbol refers to position 3, the singly primed indicates position 4).

The diversity in the chemical environment experienced by the two caffeoyl arms, even in the more symmetric 3,5- isomer is to be ascribed to the chirality of the quinic acid residue. Furthermore, even in the 3,5- form, considering a chair conformation for the quinic acid moiety, the two substituents are oriented in completely different fashion due to the fact that when one is in axial position the other is equatorial position (see Fig. 1).

In order to have a more complete picture, also ^13^C chemical shift were assigned. Data are reported in supplementary material (Tables S1-S3).

### Di-O-Caffeoylquinic Acid Isomers Self-Association

In order to study the affinity of di-O-caffeoylquinic acids towards caffeine, the self-association properties in solution have to be characterized, in order to rule out its contributions to NMR parameters.

While the entity of the phenomenon is relatively easy to study (and it is typically done following the dependence of chemical shift on concentration), its interpretation requires the choice of an appropriate model. In the case of association driven by stacking, the isodesmic model is often used, in the assumption that the association constant K describing each stacking interaction does not depend on the number of stacked molecule in the aggregate (in other words the equilibrium constant for the formation of the A_n+1_ species from A and A_n_ is always the same, regardless the value of n). As we already did in the case of the mono-O-caffeoylquinic acid [11] we analyzed our data making use of the equations describing the trend of the chemical shift as a function of the self-association in the isodesmic model assumption:

1 $$ {\updelta}^{obs}={\updelta}^m+{\varDelta}_0K\left[C\right]\left[2-K\left[C\right]\right] $$

2 $$ \left[C\right]=C{\left[\frac{2}{1+\sqrt{4KC+1}}\right]}^2 $$

where δ_m_ is the chemical shift of the monomer, ∆0 is the limiting deviation of the chemical shift of aggregate from δm (=δaggr-δm; with δaggr being the chemical shift of the fully aggregated species), [C] is the equilibrium concentration of the monomer and C the global concentration of the species. Using the shifts of protons experiencing substantial dependence on concentration, we obtained the values in Table 1.

**Table 1:** Self-association constant, K, of di-O-caffeoylquinic acid isomers (3,5– , 3,4- and 4,5-) according to the isodesmic model. Values were calculated by fitting ^1^H chemical shift deviations of protons at increasing concentration with equations (1) and (2). The error is expressed as the standard deviation of the value obtained by multiple signals

Compound	K (M^−1^)
3,5	4 ± 2
3,4	36 ± 2
4,5	63 ± 10

The constant determined for the di-O-caffeoylquinic acid isomers is on average higher than that reported for 5-O-caffeoylquinic acid (K = 2.8 ± 0.6 M^−1^) and obtained under similar experimental conditions [11]. This finding is expected in view of the increased number of aromatic rings, however, it has to be noted that the three isomers show a very different propensity to self-associate. In particular, 3,5-di-O-caffeoylquinic acid in aqueous solution shows a slightly lower tendency to aggregation if compared with the other two isomers **3** and **4** and the value of determined constant is very similar to that of the mono-caffeoylquinic acid previously studied [11]. This may indicate self-stacking, canceling the enhanced tendency, observed in the other isomers, to associate with other molecules. Differently 3,4-di-O-caffeoylquinic and 4,5-di-O-caffeoylquinic acids are characterized by a stronger self-association with constants falling within the range reported for polyphenols such as (-)-epicatechin and other tea polyphenols in aqueous media [21].

### Interaction with Caffeine

Given the values found for the self-association constants of the three compounds, we decided to study the interaction with caffeine (**5** in Scheme 1) keeping the concentration of di-O-caffeoylquinic acids constant. The self-association constant of caffeine (7.6 ± 0.5 M^−1^) [11] is in fact much lower than that of 3,4- and 4,5- isomers and comparable to that of the 3,5- isomer. Since we are studying relatively weak interactions, by the end of the titration one compound must be in high excess (at least 10 times); this means that in order to minimize interferences from self-association the compound with higher association propensity (in our case the di-O-caffeoylquinic acid) must be kept at low and constant concentration. We used a solution 1 mM for all the compounds studied. At this concentration, while the quality of the spectra is very good, the chemical shift is still relatively unchanged with respect to what observed at 1 μM where the species are completely monomeric.

**Fig. 2:**
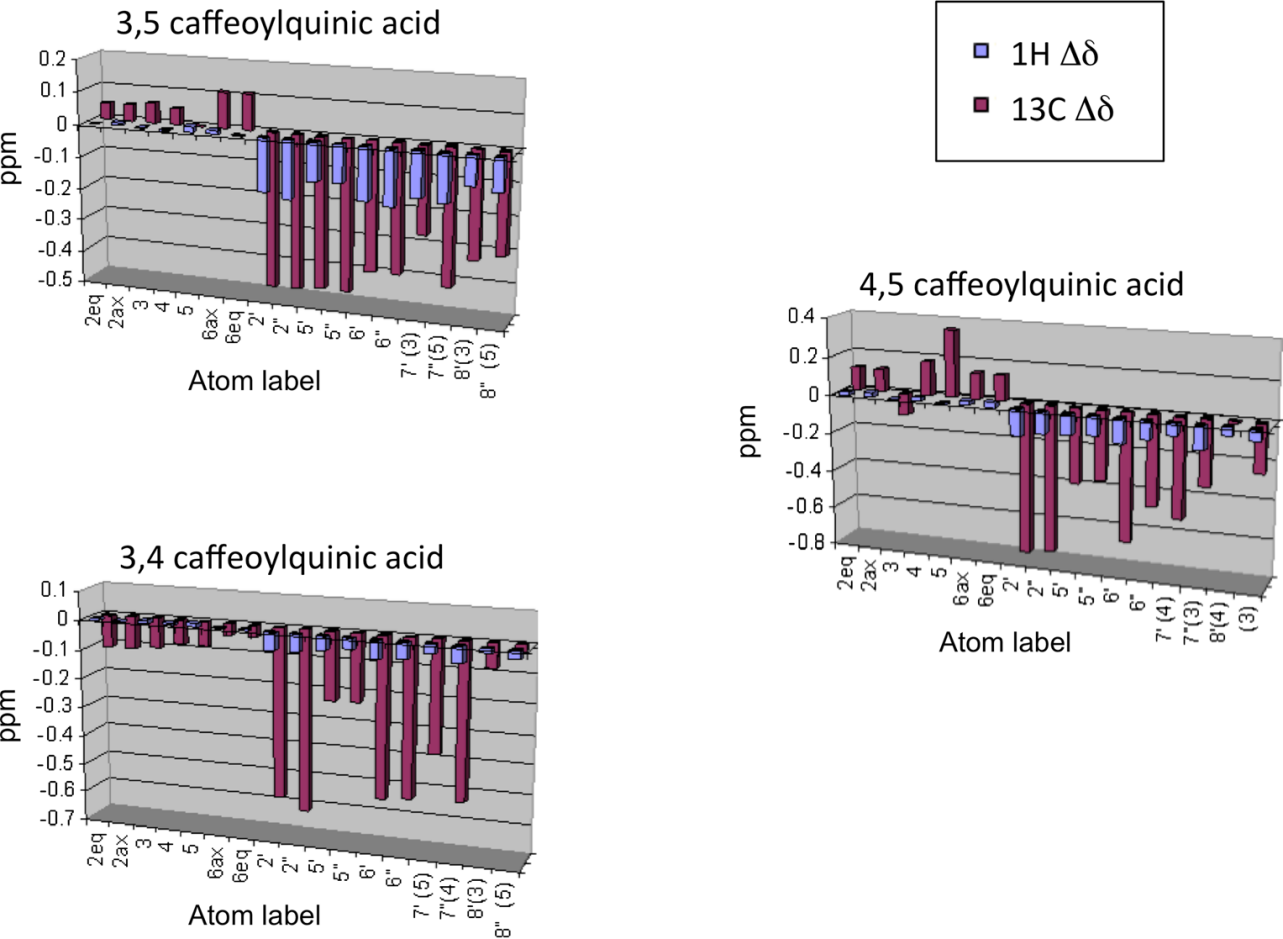
^1^H and ^13^C chemical shift deviations (∆δ) of 3,5– 4,5- and 3,4-di-O-caffeoylquinic acids upon addition of equimolar quantity of caffeine (both species 17 mM). Atom numbering as in Fig. 1

Titration of 1 mM solutions at pH 7 with concentrated caffeine (whose microliter-range addition minimize changes of di-O-caffeoylquinic concentration) cause significant shifts of virtually all signals (Fig. 2) indicating the formation of one or more complexes. In all cases shielding of the aromatic arms is apparent, strongly suggesting stacking of caffeine onto the caffeoyl aromatic plane conjugated with the double bond. Remarkably, the behavior of both arms in all compounds is very similar, suggesting that the two caffeoyl moieties experience similar chemical environment.

The equilibrium constant for the formation of the caffeine-bound species can be calculated using the following expression [11]:

3 $$ \varDelta ={\varDelta}_0\frac{{\displaystyle {C}_M^{free}}}{K_d+{C}_M^{free}} $$

where Kd is the inverse of the association constant for the formation of a M-L complex starting from M and L molecules, ∆ is the actual change of chemical shift of L (= di-O-caffeoylquinic acid), ∆_0_ is its limiting value when fully complexed and C_M_^free^ is the experimental caffeine concentration corrected by using the relation [22, 23]:

4 $$ {C}_M^{free}={C}_M-{C}_L\frac{\varDelta }{\varDelta_0} $$

where C_M_ and C_L_ are the total concentrations of caffeine and di-O-caffeoylquinic acid, respectively.

### Caffeine-3,5-di-O-Caffeoylquinic Acid Complex

**Fig. 3:**
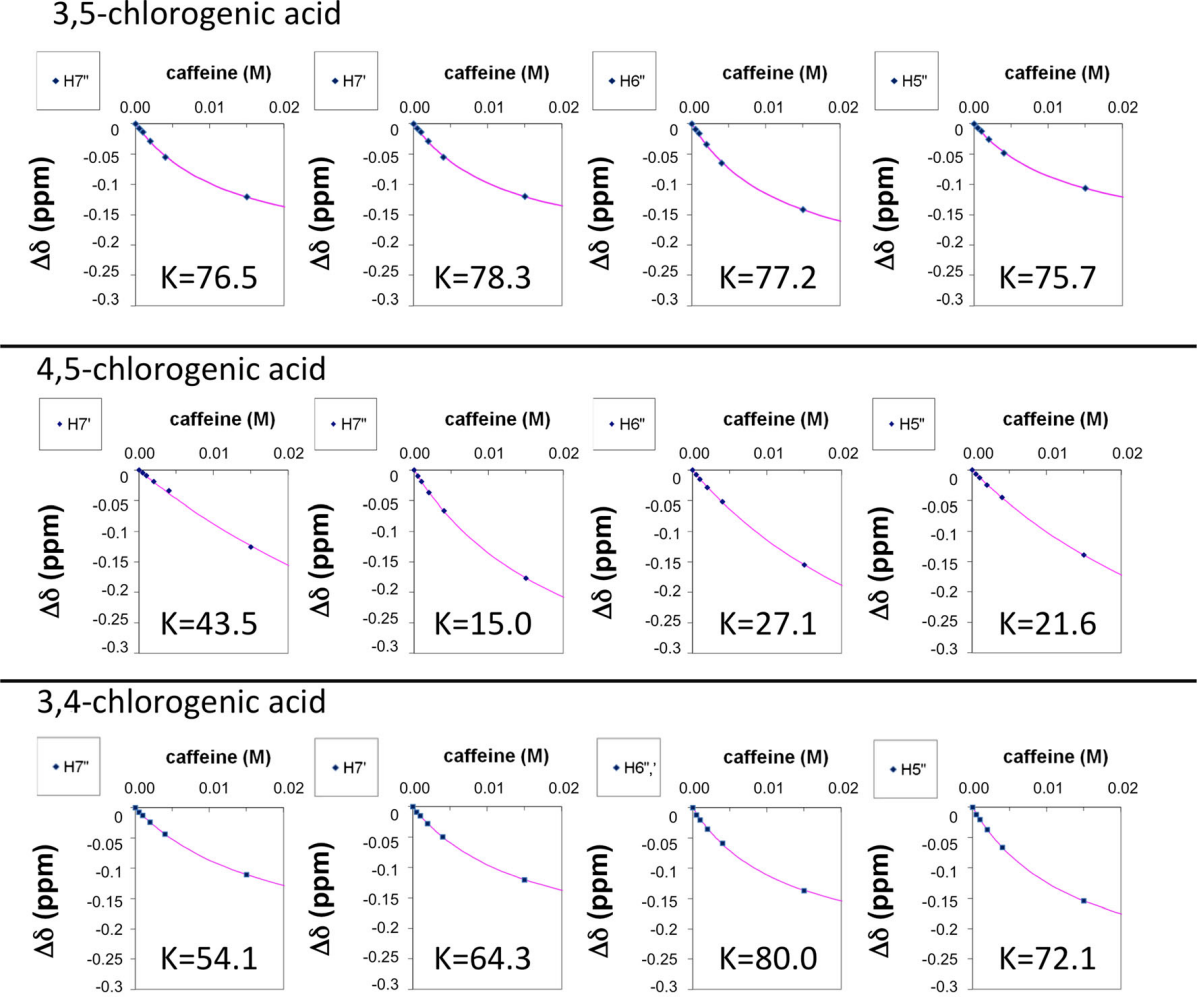
Association constants K of di-O-caffeoylquinic acids (4,5– 3,5- and 3,4-) with caffeine. Values were calculated by fitting ^1^H chemical shift deviations of different protons upon addition of increasing concentration of caffeine with equations (3) and (4). Only *curves* of protons H7’, H7”, H5” and H6” are shown for simplicity. The complete set of data is reported in Table 2

The titration curve reporting the chemical shift of di-O-caffeoylquinic acid with increasing amount of caffeine and its standard regression analysis using Eqs. (4) and (5) is reported in Fig. 3. The analysis was repeated using the chemical shift of many different protons experiencing perturbation. The complete set of measurements is reported in Table 2. We found an equilibrium constant of 78 ± 2 M^−1^, much larger than the one measured for the mono-substituted form of caffeoylquinic acid (30 ± 4 M^−1^) [11]. In the titration, caffeine was added to a maximum final concentration of 20 mM as its tendency to self-aggregation would have interfered with the measurement; this did not allow to reach the full saturation of the di-O-caffeoylquinic acid. However, the fitting curves obtained by all protons experiencing a significant perturbation of their chemical shift upon addition of caffeine, display little error and the value of the association constant derived from different protons is remarkably similar.

**Table 2:** Association constants K of di-O-caffeoylquinic acids (4,5– 3,5- and 3,4-) with caffeine. Values were calculated by fitting ^1^H chemical shift deviations of different protons upon addition of increasing concentration of caffeine with equations (3) and (4)

K (M^−1^)	H2’	H5’	H6’	H7’	H8’	H2”	H5”	H6”	H7”	H8”
4,5	25.4	30.9	35.4	15.0	26.7	23.2	21.6	27.1	45.3	32.5
3,5	78.6	77.3	79.0	78.3	81.7	76.7	75.7	77.2	76.5	77.6
3,4	64.7	84.7	80.0*	64.3	113.2	54.2	72.1	80.0*	54.1	80.6

*H6’ and H6” overlap

These observations clearly indicate that we are monitoring either a single process or multiple interactions with very similar thermodynamic properties.

In other words, the enhancement of the equilibrium constant from the mono-substitution (5-chlorogenic acid (=5-O-caffeoylquinic), Ka = 30 ± 4 M^−1^) to the di-substitution (3,5-di-O-caffeoylquinic acid, Ka = 78 ± 2 M^−1^) can be explained in two ways:

i) the presence of two independent sites (rather than one) on di-substituted quinic acid with similar affinity for caffeine (and association constant similar to chlorogenic acid) makes the sequestering of caffeine more efficient;
ii) a cooperative process creates a 1:1 complex with favored thermodynamics.

In the first case one should roughly expect that the measured equilibrium constant for the di-O-caffeoyl species K^bi^ is twice as much that found for the mono-substituted chlorogenic acid K^mono^. In fact the same number of caffeine sites is obtained with only the half of chlorogenic acid concentration. Thus, as K^mono^ = [complex]/([caffeine] [chlorogenic]), [cholorogenic] should be substituted by [3,5-di-O-caffeoyl]/2 yielding K^bi^ = K^mono^*2.

Our measured equilibrium constant for the caffeine-3,5-di-O-caffeoylquinic acid complex is actually more than twice that found for the mono-substituted acid (78 ± 2 M^−1^ *vs* 30 ± 4 M^−1^); this might indicate the formation of a much more stable 1:1 complex which could in principle harbour caffeine into the two caffeoyl arms of the 3,5-di-O-caffeoyl acid in a sandwich-like fashion. A conformation of 3,5-di-O-caffeoylquinic acid with the two caffeoyl arms facing each other has been proposed in the absence of caffeine, demonstrating that the two arms can sterically come close [13].

### Caffeine-3,4- and -4,5-di-O-Caffeoylquinic Acids Complexes

In the case of 3,4- and 4,5-di-O-caffeoylquinic acids the situation is quite different. Although the proton signals of these compounds change significantly with increasing concentration of caffeine, regression analysis of the curves by equations (4) and (5) indicates a complex behavior with different protons sensing different equilibrium constants, much more different than the fitting error. Clearly, multiple processes are present at the same time. It is important to point out that the observed chemical shift displacement in case of multiple equilibria is the result of the weighed sum of the contribution to the chemical shift from each kind of complex. The study of caffeine interacting with mono-caffeoylquinic acid has demonstrated that the stacking interaction shields all protons of the caffeoyl arm [11]. The global effect on our experimental data is very similar; Fig. 2 clearly shows a marked shielding effect on all the caffeoyl ^1^H and ^13^C chemical shifts upon hetero-association with caffeine. We can therefore safely assume that the presence of multiple stacking processes results in a “measured” equilibrium constant larger than that of each single process (we could not affirm the same for the fitting curves obtained with the protons of the quinic ring, where de-shielding is sometimes observed depending on the orientation of caffeine). In other words, as the chemical shift displacements can only cooperate in one direction (shielding effect) on the caffeoyl moiety, the chemical shift change “faster”, pushed by multiple equilibria at the same time, and finally yields a larger association constant. This also means that the lowest value of association constant obtained within a region of the molecule, is the best candidate for the description of the single interaction process.

While the global equilibrium constant is magnified by the interference among different equilibria, the actual constant for each single process could be underestimated, because of partial sequestration of the free ligand by the other sites. In this situation, one should find, for each complex, a signal whose chemical shift is unaffected by the other complexes; this signal would yield the apparent equilibrium constant. Once all the apparent equilibrium constants are determined, their value should be corrected for the mutual competition for the free ligand. Although this is impossible without the a priori knowledge of the structure of the complex, one can try to at least derive some conclusions from the abundant number of data measured.

As we just stated, the lower equilibrium constant measured within a molecular region, is the best candidate for the estimation of a single complexation process. For example in the case of 4,5-di-O-caffeoylquinic acid, the value of 15 M^−1^ found for H_7_’ is likely to give a good estimate for a complex involving the caffeoyl arm in position 5. However, other protons of the same arm seem to be affected by at least another process in their surrounding, as the value of the association constant derived from their curves is much higher (H_8_’ gives 27 M^−1^, H_2_’ gives 25 M^−1^, H_6_’ gives 35 M^−1^, H_5_’ gives 31 M^−1^). The same considerations hold for the remaining caffeoyl arm.

Quite interestingly, the value of the measured equilibrium constant in such complex system can give structural information. In fact, while it is not possible that an interaction affecting the chemical shift of a proton does not affect that of a nearby atom, it is highly likely that one proton is affected by multiple equilibria contributing cooperatively and leading to a magnified equilibrium constant. As a consequence, nearby nuclei are likely to experience the same combinations of association constants. A striking confirmation of this effect is given by the values of the association constant obtained by H_5_ and H_6_ which are forcibly close in space (they belong to the same ring): in all caffeoyl moieties of all compounds studied the value of the equilibrium constant obtained by this two signals is very similar. This concept can be applied to more interesting cases, like the distance between H_2_ and H_8_ in the caffeoyl ring. The co-planarity required for electron delocalization between the double bond and the aromatic ring in the caffeoyl arm restricts the number of favorable conformations of this moiety to two: either H_2_ or H_6_ come close to H_8_ (at 0.24 nm, the other nucleus being at 0.48 nm). Coming to our data, the similar equilibrium constant values obtained by H_2_’ and H_8_’ protons in the 4,5-di-O-caffeoylquinic acid suggest that in the complex these two protons are close in space while the opposite holds for the other (doubly primed) arm. Overlap between H_6_’ and H_6_” makes this comparison harder in the 3,4-di-O-caffeoylquinic compound, while the evaluation cannot be applied to the 3,5-di-O-caffeoylquinic acid (where equilibrium constants are not differentiated).

### General Remark on the NMR Data

NOESY spectra of solution of caffeine equimolar with each of the studied compound confirmed the assumed inter-aromatic stacking interaction. The three methyl of caffeine display NOESY and ROESY cross peaks with both the aromatic arms of all di-O-caffeoylquinic acid isomers. Though weak, these correlations are not visible with the other protons (e.g. the protons of the quinic acid ring). While we could not find intra-molecular cross peak connecting the two caffeoyl arms (at least judging from the signals of the double bond, as the aromatic are to close to the diagonal to be resolved if present), some other intra-molecular NOEs are useful in defining the conformation of the caffeoyl arms. As stated above, the relative distance between H_8_ and H_2_ (or H_6_) define the orientation of the aromatic ring with respect to the double bond. Both correlations are clearly visible in all spectra with different relative intensities. Given the sixth power dependence of NOE on the inter-nuclear distance, the volume of the NOE with the proton at largest distance is negligible compared to the one at short distance. The presence of both cross peaks at comparable intensity therefore indicates that both conformations are possible. Specifically, the conformation bringing H_2_ close to H_8_ is slightly preferred in all compounds (see Table 3). The values in Table 3 reflect the global population of the conformations independently if they are involved in the complex or refer to the free state.

**Table 3:** Percental population of isomers whose H_2_-H_8_ distance is closer than H_2_-H_6_ distance in the caffeolyl moiety

isomer	3,5	4,5	3,4
H_2_’,H_8_’/H_2_”,H_8_”	54.1/65.7	60.9/67.2	55.0/54.2

As for intermolecular NOEs, cross peaks are present connecting H_8_ (and to a lesser extent H7) with all caffeine methyl but in particular H_12_ of caffeine (this is observed for 3,5- and 4,5- compound and similar contacts were observed in the chrorogenic acid interacting with caffeine, [11]) suggesting that the caffeine approaches the chlorogenic acid with its five membered ring. In the case of 3,4-di-O-caffeoylquinic, NOEs seem to connect H_8_ to H_10_ and H_11_, as if in this case the caffeine was rotated by 180° around the axis orthogonal to the chlorogenic aromatic plane.

However, care has to be taken in interpreting these data as spin diffusion cannot be ruled out (the long mixing time used of 400 ms is necessary to reinforce weak intermolecular NOE cross peaks). Moreover both orientations can be present in different kind of complexes.

### A Structural Model of Caffeine- di-O-caffeoylquinic Acid Isomers Complexes

**Fig. 4:**
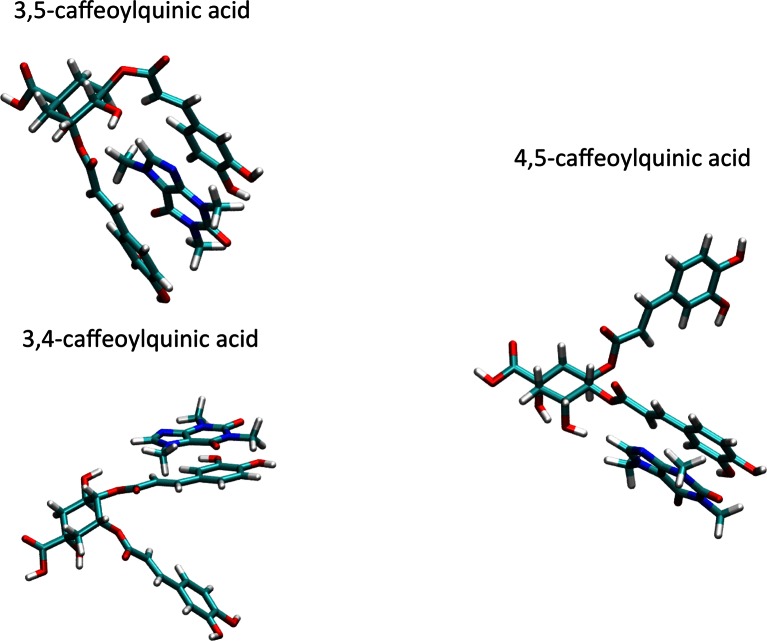
Structural models of di-O-caffeoylquinic acids (4,5– 3,5- and 3,4-) complexed with caffeine, as obtained by quantum chemical calculations

The NMR data show a consistent shielding of caffeolyl arms (Fig. 2). This strongly suggests aromatic stacking with caffeine in such complex. In addition, NOE data suggest that for the 3,5- and 4,5- isomers (but possibly also 3,4-), caffeine approaches the caffeoyl- arm with its five membered ring. Next, these data suggest a preferred orientation of the aromatic ring with respect to its double bond, as the H_2_-H_8_ distance appears on average closer than that between H_6_ and H_8_ (Table 3). Finally, the discontinuous values of the equilibrium constants measured from different protons indicate the presence of multiple equilibria between species (see Fig. 3, Table 2). Taken all of these results together, we may expect an ensemble of conformations of the complexes in solution, characterized with different orientations of the caffeine with respect to the di-O-caffeoylquinic acid. Out of many conformations calculated (figure S1), we show one most representative for each complex, obtained by quantum chemistry calculations (Fig. 4). These structures are compatible with the above experimental facts.

## Conclusions

As already observed in the case of other coffee compounds [11, 24], di-O-caffeoylquinic acid isomers are prone to interact with caffeine in aqueous solution by complex formation and then may be involved in caffeine binding observed in coffee beverages [11]. However, differently from the complex formed by caffeine with 5-caffeoylquinic acid, di-O-caffeoylquinic acid isomers in their interaction with caffeine show a complexity of multiple equilibria which makes very difficult, if not impossible, the determination of equilibrium constants. Only in the case of 3,5-di-O-caffeoylquinic acid a well definite constant can be measured. This isomer is characterized by a low tendency to self-associate and its interaction with caffeine indicates that a sandwich structure (caffeine between the two caffeoyl arms) is the most likely conformation prevailing in aqueous solution. In spite of difficulties in determining the equilibrium constants, detailed analysis of data reveal at least qualitatively the nature of the interactions taking place in solution for 4,5- and 3,4-di-O-caffeoylquinic acids interacting with caffeine. In both cases the sandwich structure seems much more unlikely. In 4,5-di-O-caffeoylquinic acid both caffeoyl arms are bound in equatorial position and this geometry forces the two aromatic plane in an orthogonal orientation with respect to the plane of the quinic saturated ring, in order to minimize steric clashes (see Fig. 4). In this case the caffeine ring is more likely to approach each caffeolyl from the external sides, causing the deshielding of most aliphatic protons that is observed by NMR (Fig. 2). Finally, the case of 3,4-di-O-caffeolilquinic is unique. In this case all protons of the saturated ring are shielded indicating that, while interacting with the caffeoyl arm, the caffeine is also capping the saturated ring (Fig. 4).

It can be hypothesized that caffeine-3,5-di-O-caffeoylquinic acid complex in aqueous solution is more conformationally stable if compared with the complexes formed by caffeine with other two isomers. It has to be stressed out that by investigating coffee chlorogenic acids biosynthesis pathway a different behavior has been observed for 3,5-di-O-caffeoylquinic acid when compared with that of the other two isomers [25]. In order to interpret this difference, the Authors suggested a possible complex formation with caffeine in the case of 3,5-di-O-caffeoylquinic acid, the other two isomers being either not interacting or complexing “at a much lower rate” [25]. The scenario emerging from the present work seems to corroborate this view. Last but not least, is the role that is played by these complexes in the physiological effects of caffeine. As the roasting process determines the relative amount of chlorogenic acid isomers [26], it cannot be excluded a priori that the different extent of caffeine complexation may contribute in determining the different biological activity displayed by coffee at different roasting degree [27, 28].

## Electronic supplementary material

Below is the link to the electronic supplementary material.

Figure S1 (DOCX 24347 kb)

Table S1 (DOCX 25 kb)

Table S2 (DOCX 25 kb)

Table S3 (DOCX 25 kb)

## Abbreviations

HSQC: Heteronuclear Single Quantum Coherence
NOE: Nuclear Overhauser Effect
NOESY: Nuclear Overhauser Effect SpectroscopY
ROESY: ROtating frame Overhauser SpectroscopY
TOCSY: TOtal Correlation SpectroscopY
