# Supplementary material for: NMR Studies of Hetero-Association of Caffeine with di-O-Caffeoylquinic Acid Isomers in Aqueous Solution
Source: Food Biophys. 2014 Oct 3;10(3):235–43. doi: 10.1007/s11483-014-9368-x (PMC4512271; doi:10.1007/s11483-014-9368-x)

**Figure S1.** *Structural models of di-O-caffeoylquinic acids (4,5- 3,5- and 3,4-) complexed with caffeine, in different relative orientations compatible with NMR data.*


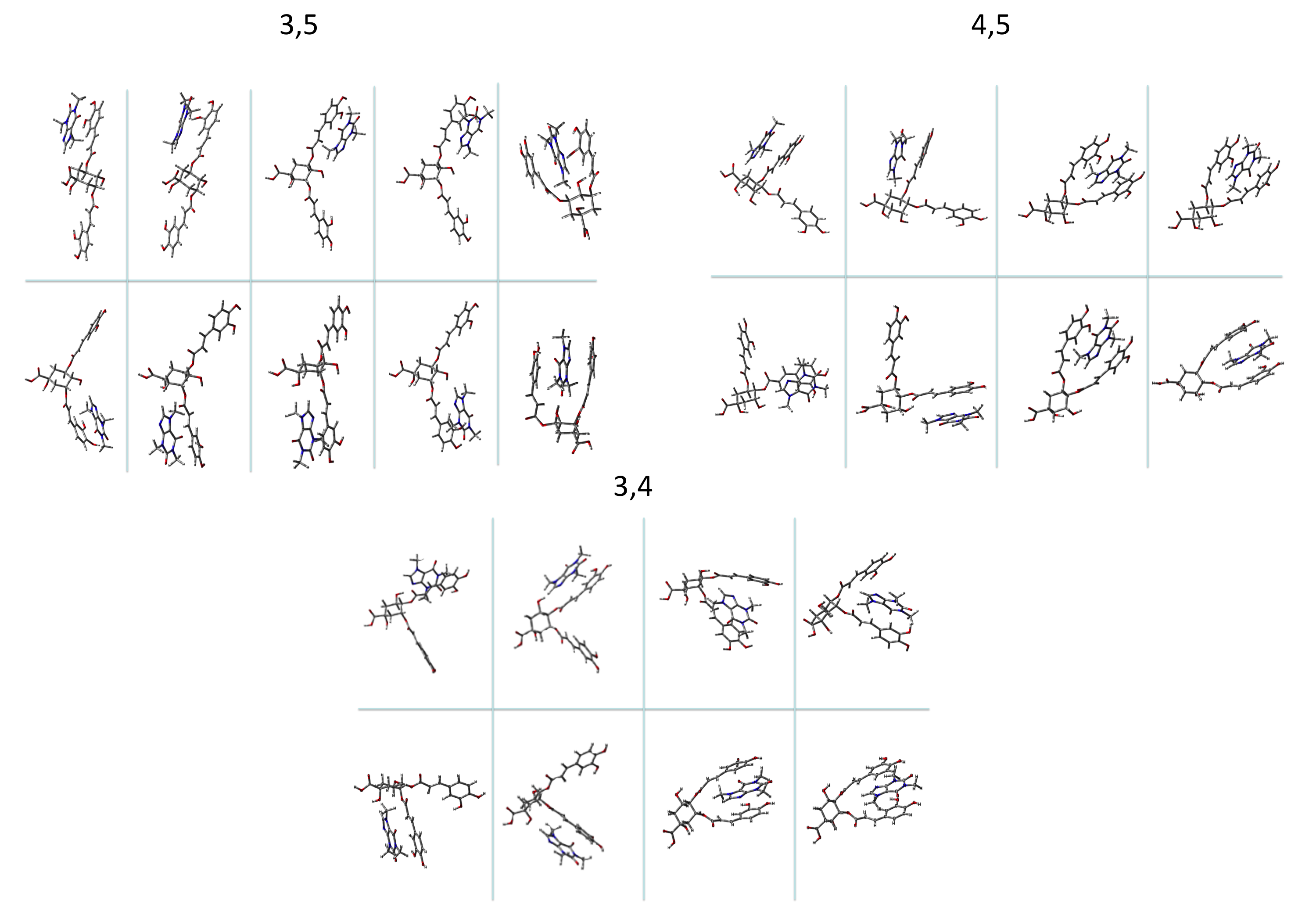

Supplement: Supplementary file 1 — (DOCX 24347 kb) [file 11483_2014_9368_MOESM1_ESM.docx]
