# Supplementary material for: NMR Studies of Hetero-Association of Caffeine with di-O-Caffeoylquinic Acid Isomers in Aqueous Solution
Source: Food Biophys. 2014 Oct 3;10(3):235–43. doi: 10.1007/s11483-014-9368-x (PMC4512271; doi:10.1007/s11483-014-9368-x)
Supplement: Supplementary file 2 — (DOCX 25 kb) [file 11483_2014_9368_MOESM2_ESM.docx]

**Table S1. ^1^H and ^13^C chemical shifts in 4,5 Chlorogenic acid 20mM.** *Values are referenced to internal TSP. The solution was buffered at pH 7 with phosphate 80mM. Atoms labelled by ‘ likely belong to the arm attached in position 5 while those labelled by “ to that in position 4.*

| **position** | **^1^H chemical shift (ppm)** | **Calculated** | **^13^C chemical shift (ppm)** | **Calculated** |
| --- | --- | --- | --- | --- |
| 2eq | 2.23 | 2.4 | 40.4 | 42 |
| 2ax | 2.10 | 3.79 | 40.4 | 42 |
| 3 | 5.66 | 4.83 | 69.1 | 75.6 |
| 4 | 5.01 | 6.29 | 75.3 | 72.7 |
| 5 | 4.44 | 4.55 | 64.7 | 72.9 |
| 6ax | 2.38 | 2.51 | 35.5 | 40.4 |
| 6eq | 2.10 | 2.51 | 35.5 | 40.4 |
| 2' | 6.90 | 7.02 | 114.8 | 122 |
| 2” | 6.97 | 7.17 | 115.0 | 121.9 |
| 5' | 6.67 | 7.17 | 115.7 | 117.9 |
| 5” | 6.81 | 7.1 | 115.8 | 117.9 |
| 6' | 6.69 | 7.91 | 122.7 | 121 |
| 6” | 6.83 | 7.77 | 122.5 | 120.6 |
| 7' | 7.37 | 7.91 | 146.8 | 152.7 |
| 7” | 7.41 | 7.91 | 146.6 | 150.5 |
| 8' | 6.16 | 6.73 | 113.2 | 114.6 |
| 8” | 6.34 | 6.58 | 114.0 | 116.9 |
