# Supplementary material for: NMR Studies of Hetero-Association of Caffeine with di-O-Caffeoylquinic Acid Isomers in Aqueous Solution
Source: Food Biophys. 2014 Oct 3;10(3):235–43. doi: 10.1007/s11483-014-9368-x (PMC4512271; doi:10.1007/s11483-014-9368-x)
Supplement: Supplementary file 3 — (DOCX 25 kb) [file 11483_2014_9368_MOESM3_ESM.docx]

**NMR Table S2. ^1^H and ^13^C chemical shifts in 3,5 Chlorogenic acid 20mM.** *Values are referenced to internal TSP. The solution was buffered at pH 7 with phosphate 80mM. Atoms labelled by ‘ likely belong to the arm attached in position 5 while those labelled by “ to that in position 3.*

| **position** | **^1^H chemical shift (ppm)** | **Calculated** | **^13^C chemical shift (ppm)** | **Calculated** |
| --- | --- | --- | --- | --- |
| 2eq | 2.23 | 2.22 | 41.4 | 42.7 |
| 2ax | 2.09 | 2.33 | 41.4 | 42.7 |
| 3 | 5.49 | 5.73 | 76.0 | 76.7 |
| 4 | 4.10 | 4.32 | 74.4 | 77.7 |
| 5 | 5.50 | 5.63 | 73.8 | 78 |
| 6ax | 2.31 | 2.45 | 38.7 | 43.7 |
| 6eq | 2.15 | 2.33 | 38.7 | 43.7 |
| 2' | 7.13 | 6.9 | 118.1 | 120.5 |
| 2” | 7.17 | 7.18 | 118.1 | 112.9 |
| 5' | 6.91 | 6.9 | 119.3 | 116.6 |
| 5” | 6.92 | 7.26 | 119.3 | 115.1 |
| 6' | 7.05 | 7.72 | 125.7 | 121.2 |
| 6” | 7.09 | 7.48 | 125.7 | 131.8 |
| 7' | 7.59 | 8.66 | 149.2 | 152.4 |
| 7” | 7.63 | 7.88 | 149.4 | 155.1 |
| 8' | 6.32 | 6.37 | 117.5 | 115.2 |
| 8” | 6.43 | 6.13 | 117.7 | 112.6 |
