# Supplementary material for: NMR Studies of Hetero-Association of Caffeine with di-O-Caffeoylquinic Acid Isomers in Aqueous Solution
Source: Food Biophys. 2014 Oct 3;10(3):235–43. doi: 10.1007/s11483-014-9368-x (PMC4512271; doi:10.1007/s11483-014-9368-x)
Supplement: Supplementary file 4 — (DOCX 25 kb) [file 11483_2014_9368_MOESM4_ESM.docx]

**Table S3. ^1^H and ^13^C chemical shifts in 3,4 Chlorogenic acid 20mM.** *Values are referenced to internal TSP. The solution was buffered at pH 7 with phosphate 80mM. Atoms labelled by ‘ likely belong to the arm attached in position 4 while those labelled by “ to that in position 3*

| **position** | **^1^H chemical shift (ppm)** | **Calculated** | **^13^C chemical shift (ppm)** | **Calculated** |
| --- | --- | --- | --- | --- |
| 2eq | 2.30 | 2.55 | 38.8 | 41.9 |
| 2ax | 2.22 | 2.38 | 38.8 | 41.9 |
| 3 | 4.46 | 4.65 | 68.8 | 66 |
| 4 | 5.17 | 4.91 | 75.8 | 79.7 |
| 5 | 5.65 | 5.96 | 68.4 | 68.6 |
| 6ax | 2.30 | 2.23 | 37.3 | 42.3 |
| 6eq | 2.14 | 2.38 | 37.3 | 42.3 |
| 2' | 6.76 | 7.04 | 115.1 | 122 |
| 2” | 6.84 | 7.17 | 115.2 | 122 |
| 5' | 6.67 | 7.17 | 115.8 | 118 |
| 5” | 6.68 | 7.17 | 115.8 | 118 |
| 6' | 6.59 | 7.9 | 122.4 | 120.8 |
| 6” | 6.69 | 7.81 | 122.5 | 120.7 |
| 7' | 7.25 | 7.9 | 146.5 | 151.1 |
| 7” | 7.39 | 8 | 146.9 | 151.1 |
| 8' | 5.95 | 6.74 | 113.6 | 115.8 |
| 8” | 6.12 | 6.63 | 113.3 | 116 |
